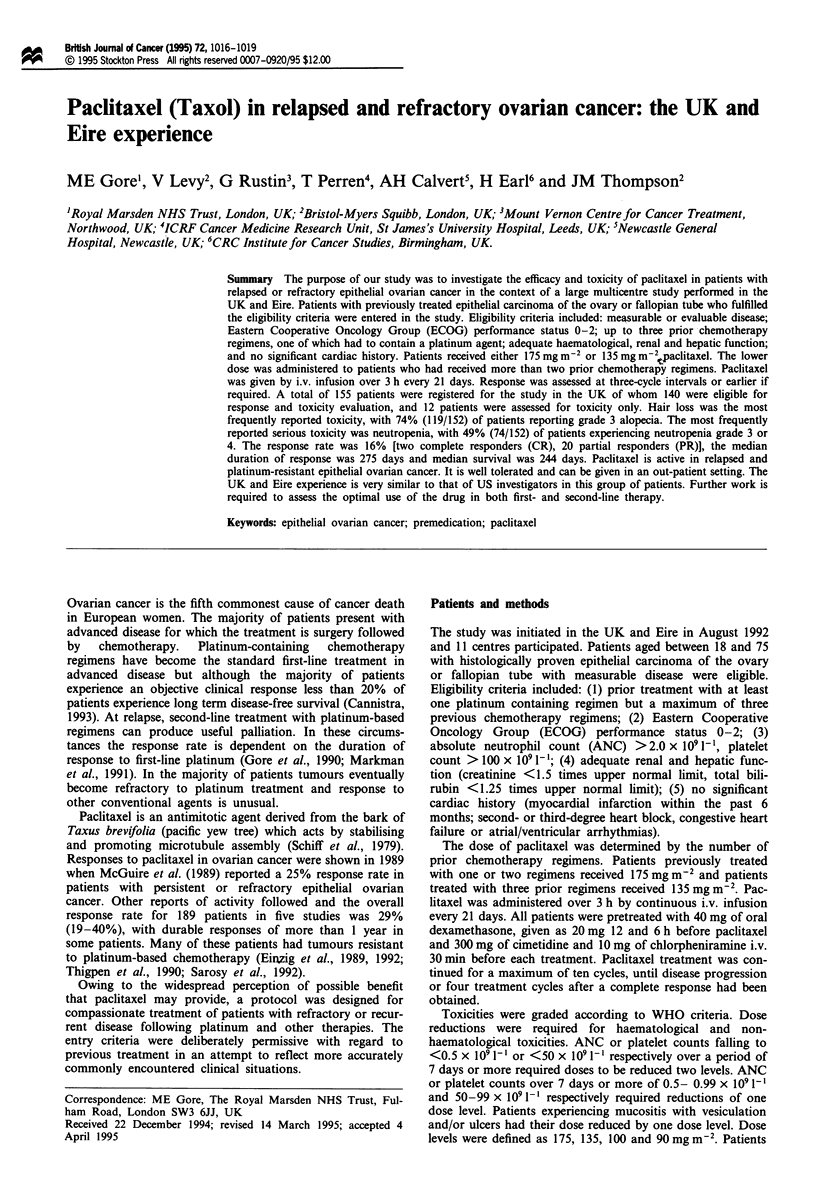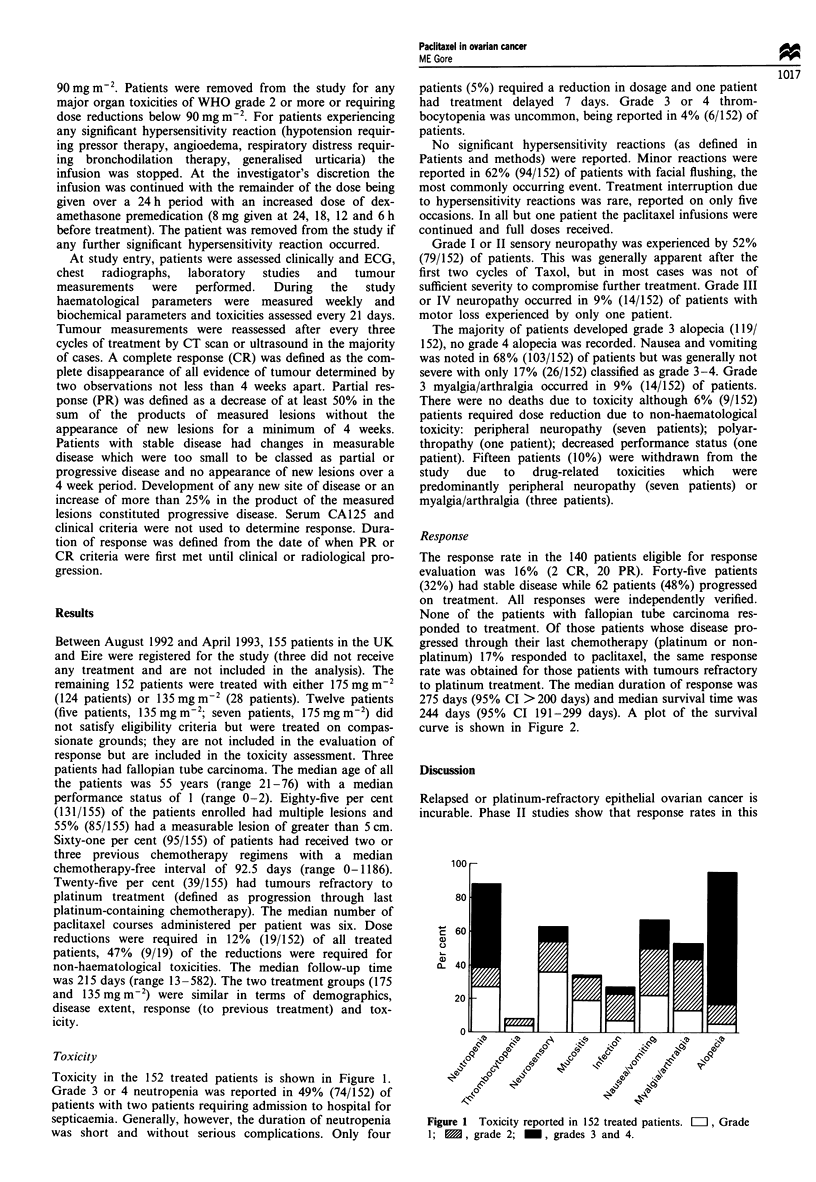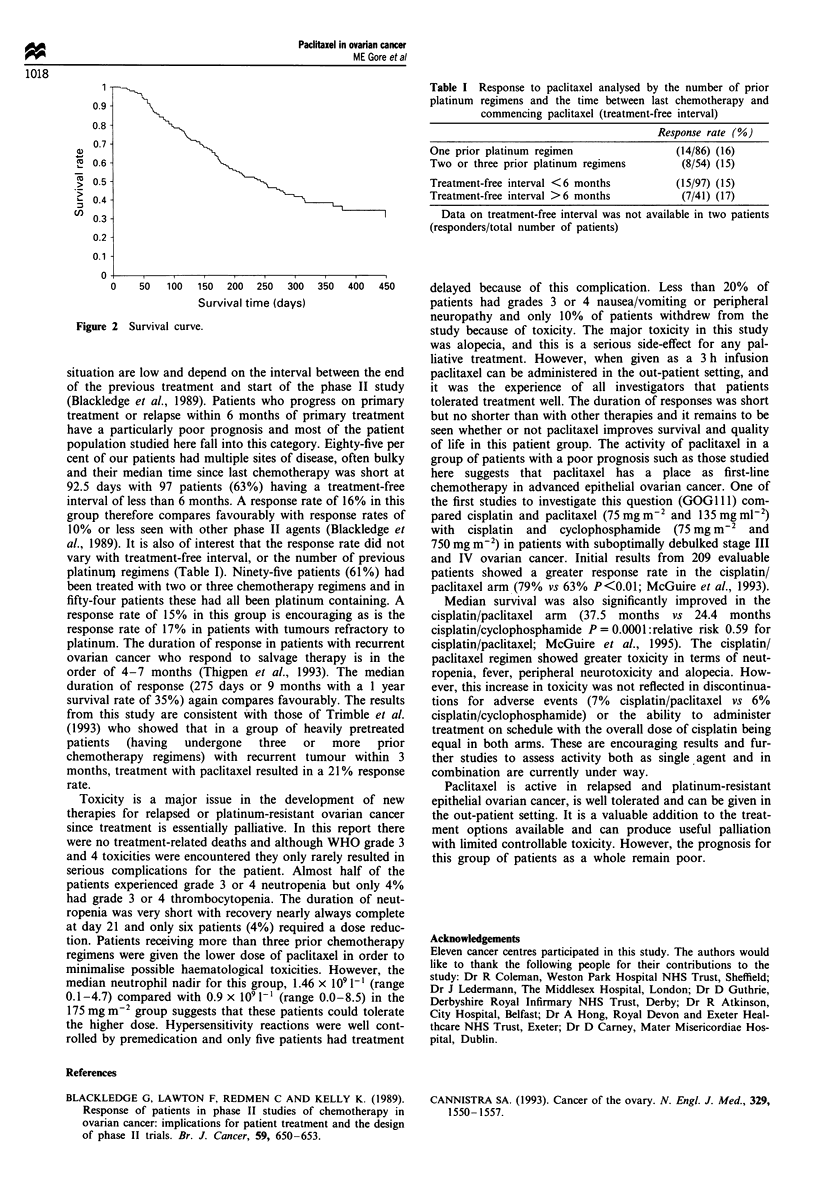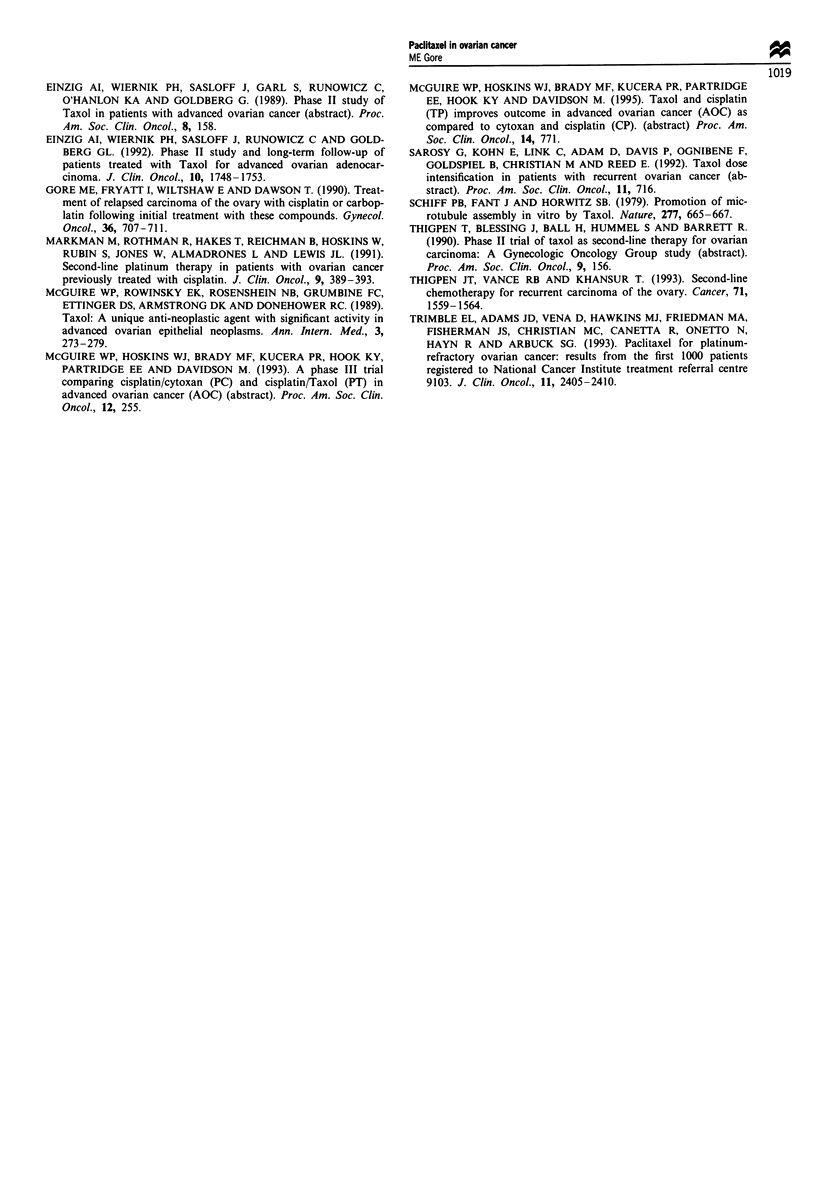# Paclitaxel (Taxol) in relapsed and refractory ovarian cancer: the UK and Eire experience.

**DOI:** 10.1038/bjc.1995.453

**Published:** 1995-10

**Authors:** M. E. Gore, V. Levy, G. Rustin, T. Perren, A. H. Calvert, H. Earl, J. M. Thompson

**Affiliations:** Royal Marsden NHS Trust, London, UK.

## Abstract

The purpose of our study was to investigate the efficacy and toxicity of paclitaxel in patients with relapsed or refractory epithelial ovarian cancer in the context of a large multicentre study performed in the UK and Eire. Patients with previously treated epithelial carcinoma of the ovary or fallopian tube who fulfilled the eligibility criteria were entered in the study. Eligibility criteria included: measurable or evaluable disease; Eastern Cooperative Oncology Group (ECOG) performance status 0-2; up to three prior chemotherapy regimens, one of which had to contain a platinum agent; adequate haematological, renal and hepatic function; and no significant cardiac history. Patients received either 175 mg m-2 or 135 mg m-2 paclitaxel. The lower dose was administered to patients who had received more than two prior chemotherapy regimens. Paclitaxel was given by i.v. infusion over 3 h every 21 days. Response was assessed at three-cycle intervals or earlier if required. A total of 155 patients were registered for the study in the UK of whom 140 were eligible for response and toxicity evaluation, and 12 patients were assessed for toxicity only. Hair loss was the most frequently reported toxicity, with 74% (119/152) of patients reporting grade 3 alopecia. The most frequently reported serious toxicity was neutropenia, with 49% (74/152) of patients experiencing neutropenia grade 3 or 4. The response rate was 16% [two complete responders (CR), 20 partial responders (PR)], the median duration of response was 275 days and median survival was 244 days. Paclitaxel is active in relapsed and platinum-resistant epithelial ovarian cancer. It is well tolerated and can be given in an out-patient setting. The UK and Eire experience is very similar to that of US investigators in this group of patients. Further work is required to assess the optimal use of the drug in both first- and second-line therapy.


British Journal of Cancer (1995) 72, 1016-1019
© 1995 Stockton Press All rights reserved 0007-0920/95 $12.00

### Paclitaxel (Taxol) in relapsed and refractory ovarian cancer: the UK and Eire experience

ME Gore[1], V Levy[2], G Rustin[3], T Perren[4], AH Calvert[5], H Earl[6] and JM Thompson[2]

[1]*Royal Marsden NHS Trust, London, UK;* [2]*Bristol-Myers Squibb, London, UK;* [3]*Mount Vernon Centre for Cancer Treatment, Northwood, UK;* [4]*ICRF Cancer Medicine Research Unit, St James's University Hospital, Leeds, UK;* [5]*Newcastle General Hospital, Newcastle, UK;* [6]*CRC Institute for Cancer Studies, Birmingham, UK.*

**Summary** The purpose of our study was to investigate the efficacy and toxicity of paclitaxel in patients with relapsed or refractory epithelial ovarian cancer in the context of a large multicentre study performed in the UK and Eire. Patients with previously treated epithelial carcinoma of the ovary or fallopian tube who fulfilled the eligibility criteria were entered in the study. Eligibility criteria included: measurable or evaluable disease; Eastern Cooperative Oncology Group (ECOG) performance status 0-2; up to three prior chemotherapy regimens, one of which had to contain a platinum agent; adequate haematological, renal and hepatic function; and no significant cardiac history. Patients received either 175 mg m$^{-2}$ or 135 mg m$^{-2}$ paclitaxel. The lower dose was administered to patients who had received more than two prior chemotherapy regimens. Paclitaxel was given by i.v. infusion over 3 h every 21 days. Response was assessed at three-cycle intervals or earlier if required. A total of 155 patients were registered for the study in the UK of whom 140 were eligible for response and toxicity evaluation, and 12 patients were assessed for toxicity only. Hair loss was the most frequently reported toxicity, with 74% (119/152) of patients reporting grade 3 alopecia. The most frequently reported serious toxicity was neutropenia, with 49% (74/152) of patients experiencing neutropenia grade 3 or 4. The response rate was 16% [two complete responders (CR), 20 partial responders (PR)], the median duration of response was 275 days and median survival was 244 days. Paclitaxel is active in relapsed and platinum-resistant epithelial ovarian cancer. It is well tolerated and can be given in an out-patient setting. The UK and Eire experience is very similar to that of US investigators in this group of patients. Further work is required to assess the optimal use of the drug in both first- and second-line therapy.

**Keywords:** epithelial ovarian cancer; premedication; paclitaxel

Ovarian cancer is the fifth commonest cause of cancer death in European women. The majority of patients present with advanced disease for which the treatment is surgery followed by chemotherapy. Platinum-containing chemotherapy regimens have become the standard first-line treatment in advanced disease but although the majority of patients experience an objective clinical response less than 20% of patients experience long term disease-free survival (Cannistra, 1993). At relapse, second-line treatment with platinum-based regimens can produce useful palliation. In these circumstances the response rate is dependent on the duration of response to first-line platinum (Gore *et al.*, 1990; Markman *et al.*, 1991). In the majority of patients tumours eventually become refractory to platinum treatment and response to other conventional agents is unusual.

Paclitaxel is an antimitotic agent derived from the bark of *Taxus brevifolia* (pacific yew tree) which acts by stabilising and promoting microtubule assembly (Schiff *et al.*, 1979). Responses to paclitaxel in ovarian cancer were shown in 1989 when McGuire *et al.* (1989) reported a 25% response rate in patients with persistent or refractory epithelial ovarian cancer. Other reports of activity followed and the overall response rate for 189 patients in five studies was 29% (19-40%), with durable responses of more than 1 year in some patients. Many of these patients had tumours resistant to platinum-based chemotherapy (Einzig *et al.*, 1989, 1992; Thigpen *et al.*, 1990; Sarosy *et al.*, 1992).

Owing to the widespread perception of possible benefit that paclitaxel may provide, a protocol was designed for compassionate treatment of patients with refractory or recurrent disease following platinum and other therapies. The entry criteria were deliberately permissive with regard to previous treatment in an attempt to reflect more accurately commonly encountered clinical situations.

Correspondence: ME Gore, The Royal Marsden NHS Trust, Fulham Road, London SW3 6JJ, UK
Received 22 December 1994; revised 14 March 1995; accepted 4 April 1995

### Patients and methods

The study was initiated in the UK and Eire in August 1992 and 11 centres participated. Patients aged between 18 and 75 with histologically proven epithelial carcinoma of the ovary or fallopian tube with measurable disease were eligible. Eligibility criteria included: (1) prior treatment with at least one platinum containing regimen but a maximum of three previous chemotherapy regimens; (2) Eastern Cooperative Oncology Group (ECOG) performance status 0-2; (3) absolute neutrophil count (ANC) $>2.0 \times 10^9 l^{-1}$, platelet count $>100 \times 10^9 l^{-1}$; (4) adequate renal and hepatic function (creatinine $<1.5$ times upper normal limit, total bilirubin $<1.25$ times upper normal limit); (5) no significant cardiac history (myocardial infarction within the past 6 months; second- or third-degree heart block, congestive heart failure or atrial/ventricular arrhythmias).

The dose of paclitaxel was determined by the number of prior chemotherapy regimens. Patients previously treated with one or two regimens received 175 mg m$^{-2}$ and patients treated with three prior regimens received 135 mg m$^{-2}$. Paclitaxel was administered over 3 h by continuous i.v. infusion every 21 days. All patients were pretreated with 40 mg of oral dexamethasone, given as 20 mg 12 and 6 h before paclitaxel and 300 mg of cimetidine and 10 mg of chlorpheniramine i.v. 30 min before each treatment. Paclitaxel treatment was continued for a maximum of ten cycles, until disease progression or four treatment cycles after a complete response had been obtained.

Toxicities were graded according to WHO criteria. Dose reductions were required for haematological and non-haematological toxicities. ANC or platelet counts falling to $<0.5 \times 10^9 l^{-1}$ or $<50 \times 10^9 l^{-1}$ respectively over a period of 7 days or more required doses to be reduced two levels. ANC or platelet counts over 7 days or more of 0.5- $0.99 \times 10^9 l^{-1}$ and $50-99 \times 10^9 l^{-1}$ respectively required reductions of one dose level. Patients experiencing mucositis with vesiculation and/or ulcers had their dose reduced by one dose level. Dose levels were defined as 175, 135, 100 and 90 mg m$^{-2}$. Patients

$90\,mg\,m^{-2}$. Patients were removed from the study for any major organ toxicities of WHO grade 2 or more or requiring dose reductions below $90\,mg\,m^{-2}$. For patients experiencing any significant hypersensitivity reaction (hypotension requiring pressor therapy, angioedema, respiratory distress requiring bronchodilation therapy, generalised urticaria) the infusion was stopped. At the investigator's discretion the infusion was continued with the remainder of the dose being given over a 24 h period with an increased dose of dexamethasone premedication (8 mg given at 24, 18, 12 and 6 h before treatment). The patient was removed from the study if any further significant hypersensitivity reaction occurred.

At study entry, patients were assessed clinically and ECG, chest radiographs, laboratory studies and tumour measurements were performed. During the study haematological parameters were measured weekly and biochemical parameters and toxicities assessed every 21 days. Tumour measurements were reassessed after every three cycles of treatment by CT scan or ultrasound in the majority of cases. A complete response (CR) was defined as the complete disappearance of all evidence of tumour determined by two observations not less than 4 weeks apart. Partial response (PR) was defined as a decrease of at least 50% in the sum of the products of measured lesions without the appearance of new lesions for a minimum of 4 weeks. Patients with stable disease had changes in measurable disease which were too small to be classed as partial or progressive disease and no appearance of new lesions over a 4 week period. Development of any new site of disease or an increase of more than 25% in the product of the measured lesions constituted progressive disease. Serum CA125 and clinical criteria were not used to determine response. Duration of response was defined from the date of when PR or CR criteria were first met until clinical or radiological progression.

### Results

Between August 1992 and April 1993, 155 patients in the UK and Eire were registered for the study (three did not receive any treatment and are not included in the analysis). The remaining 152 patients were treated with either $175\,mg\,m^{-2}$ (124 patients) or $135\,mg\,m^{-2}$ (28 patients). Twelve patients (five patients, $135\,mg\,m^{-2}$; seven patients, $175\,mg\,m^{-2}$) did not satisfy eligibility criteria but were treated on compassionate grounds; they are not included in the evaluation of response but are included in the toxicity assessment. Three patients had fallopian tube carcinoma. The median age of all the patients was 55 years (range 21–76) with a median performance status of 1 (range 0–2). Eighty-five per cent (131/155) of the patients enrolled had multiple lesions and 55% (85/155) had a measurable lesion of greater than 5 cm. Sixty-one per cent (95/155) of patients had received two or three previous chemotherapy regimens with a median chemotherapy-free interval of 92.5 days (range 0–1186). Twenty-five per cent (39/155) had tumours refractory to platinum treatment (defined as progression through last platinum-containing chemotherapy). The median number of paclitaxel courses administered per patient was six. Dose reductions were required in 12% (19/152) of all treated patients, 47% (9/19) of the reductions were required for non-haematological toxicities. The median follow-up time was 215 days (range 13–582). The two treatment groups (175 and $135\,mg\,m^{-2}$) were similar in terms of demographics, disease extent, response (to previous treatment) and toxicity.

### *Toxicity*

Toxicity in the 152 treated patients is shown in Figure 1. Grade 3 or 4 neutropenia was reported in 49% (74/152) of patients with two patients requiring admission to hospital for septicaemia. Generally, however, the duration of neutropenia was short and without serious complications. Only four patients (5%) required a reduction in dosage and one patient had treatment delayed 7 days. Grade 3 or 4 thrombocytopenia was uncommon, being reported in 4% (6/152) of patients.

No significant hypersensitivity reactions (as defined in Patients and methods) were reported. Minor reactions were reported in 62% (94/152) of patients with facial flushing, the most commonly occurring event. Treatment interruption due to hypersensitivity reactions was rare, reported on only five occasions. In all but one patient the paclitaxel infusions were continued and full doses received.

Grade I or II sensory neuropathy was experienced by 52% (79/152) of patients. This was generally apparent after the first two cycles of Taxol, but in most cases was not of sufficient severity to compromise further treatment. Grade III or IV neuropathy occurred in 9% (14/152) of patients with motor loss experienced by only one patient.

The majority of patients developed grade 3 alopecia (119/152), no grade 4 alopecia was recorded. Nausea and vomiting was noted in 68% (103/152) of patients but was generally not severe with only 17% (26/152) classified as grade 3–4. Grade 3 myalgia/arthralgia occurred in 9% (14/152) of patients. There were no deaths due to toxicity although 6% (9/152) patients required dose reduction due to non-haematological toxicity: peripheral neuropathy (seven patients); polyarthropathy (one patient); decreased performance status (one patient). Fifteen patients (10%) were withdrawn from the study due to drug-related toxicities which were predominantly peripheral neuropathy (seven patients) or myalgia/arthralgia (three patients).

### *Response*

The response rate in the 140 patients eligible for response evaluation was 16% (2 CR, 20 PR). Forty-five patients (32%) had stable disease while 62 patients (48%) progressed on treatment. All responses were independently verified. None of the patients with fallopian tube carcinoma responded to treatment. Of those patients whose disease progressed through their last chemotherapy (platinum or non-platinum) 17% responded to paclitaxel, the same response rate was obtained for those patients with tumours refractory to platinum treatment. The median duration of response was 275 days (95% CI >200 days) and median survival time was 244 days (95% CI 191–299 days). A plot of the survival curve is shown in Figure 2.

### Discussion

Relapsed or platinum-refractory epithelial ovarian cancer is incurable. Phase II studies show that response rates in this

**Figure 1** Toxicity reported in 152 treated patients. □, Grade 1; ▨, grade 2; ■, grades 3 and 4.

**Figure 2** Survival curve.

situation are low and depend on the interval between the end of the previous treatment and start of the phase II study (Blackledge *et al.*, 1989). Patients who progress on primary treatment or relapse within 6 months of primary treatment have a particularly poor prognosis and most of the patient population studied here fall into this category. Eighty-five per cent of our patients had multiple sites of disease, often bulky and their median time since last chemotherapy was short at 92.5 days with 97 patients (63%) having a treatment-free interval of less than 6 months. A response rate of 16% in this group therefore compares favourably with response rates of 10% or less seen with other phase II agents (Blackledge *et al.*, 1989). It is also of interest that the response rate did not vary with treatment-free interval, or the number of previous platinum regimens (Table I). Ninety-five patients (61%) had been treated with two or three chemotherapy regimens and in fifty-four patients these had all been platinum containing. A response rate of 15% in this group is encouraging as is the response rate of 17% in patients with tumours refractory to platinum. The duration of response in patients with recurrent ovarian cancer who respond to salvage therapy is in the order of 4–7 months (Thigpen *et al.*, 1993). The median duration of response (275 days or 9 months with a 1 year survival rate of 35%) again compares favourably. The results from this study are consistent with those of Trimble *et al.* (1993) who showed that in a group of heavily pretreated patients (having undergone three or more prior chemotherapy regimens) with recurrent tumour within 3 months, treatment with paclitaxel resulted in a 21% response rate.

Toxicity is a major issue in the development of new therapies for relapsed or platinum-resistant ovarian cancer since treatment is essentially palliative. In this report there were no treatment-related deaths and although WHO grade 3 and 4 toxicities were encountered they only rarely resulted in serious complications for the patient. Almost half of the patients experienced grade 3 or 4 neutropenia but only 4% had grade 3 or 4 thrombocytopenia. The duration of neutropenia was very short with recovery nearly always complete at day 21 and only six patients (4%) required a dose reduction. Patients receiving more than three prior chemotherapy regimens were given the lower dose of paclitaxel in order to minimalise possible haematological toxicities. However, the median neutrophil nadir for this group, $1.46 \times 10^9 \, l^{-1}$ (range 0.1–4.7) compared with $0.9 \times 10^9 \, l^{-1}$ (range 0.0–8.5) in the $175 \, mg \, m^{-2}$ group suggests that these patients could tolerate the higher dose. Hypersensitivity reactions were well controlled by premedication and only five patients had treatment

**Table I** Response to paclitaxel analysed by the number of prior platinum regimens and the time between last chemotherapy and commencing paclitaxel (treatment-free interval)

| | *Response rate (%)* |
|---|---|
| One prior platinum regimen | (14/86) (16) |
| Two or three prior platinum regimens | (8/54) (15) |
| Treatment-free interval <6 months | (15/97) (15) |
| Treatment-free interval >6 months | (7/41) (17) |

Data on treatment-free interval was not available in two patients (responders/total number of patients)

delayed because of this complication. Less than 20% of patients had grades 3 or 4 nausea/vomiting or peripheral neuropathy and only 10% of patients withdrew from the study because of toxicity. The major toxicity in this study was alopecia, and this is a serious side-effect for any palliative treatment. However, when given as a 3 h infusion paclitaxel can be administered in the out-patient setting, and it was the experience of all investigators that patients tolerated treatment well. The duration of responses was short but no shorter than with other therapies and it remains to be seen whether or not paclitaxel improves survival and quality of life in this patient group. The activity of paclitaxel in a group of patients with a poor prognosis such as those studied here suggests that paclitaxel has a place as first-line chemotherapy in advanced epithelial ovarian cancer. One of the first studies to investigate this question (GOG111) compared cisplatin and paclitaxel ($75 \, mg \, m^{-2}$ and $135 \, mg \, ml^{-2}$) with cisplatin and cyclophosphamide ($75 \, mg \, m^{-2}$ and $750 \, mg \, m^{-2}$) in patients with suboptimally debulked stage III and IV ovarian cancer. Initial results from 209 evaluable patients showed a greater response rate in the cisplatin/paclitaxel arm (79% *vs* 63% $P<0.01$; McGuire *et al.*, 1993).

Median survival was also significantly improved in the cisplatin/paclitaxel arm (37.5 months *vs* 24.4 months cisplatin/cyclophosphamide $P = 0.0001$:relative risk 0.59 for cisplatin/paclitaxel; McGuire *et al.*, 1995). The cisplatin/paclitaxel regimen showed greater toxicity in terms of neutropenia, fever, peripheral neurotoxicity and alopecia. However, this increase in toxicity was not reflected in discontinuations for adverse events (7% cisplatin/paclitaxel *vs* 6% cisplatin/cyclophosphamide) or the ability to administer treatment on schedule with the overall dose of cisplatin being equal in both arms. These are encouraging results and further studies to assess activity both as single agent and in combination are currently under way.

Paclitaxel is active in relapsed and platinum-resistant epithelial ovarian cancer, is well tolerated and can be given in the out-patient setting. It is a valuable addition to the treatment options available and can produce useful palliation with limited controllable toxicity. However, the prognosis for this group of patients as a whole remain poor.

**Acknowledgements**

Eleven cancer centres participated in this study. The authors would like to thank the following people for their contributions to the study: Dr R Coleman, Weston Park Hospital NHS Trust, Sheffield; Dr J Ledermann, The Middlesex Hospital, London; Dr D Guthrie, Derbyshire Royal Infirmary NHS Trust, Derby; Dr R Atkinson, City Hospital, Belfast; Dr A Hong, Royal Devon and Exeter Healthcare NHS Trust, Exeter; Dr D Carney, Mater Misericordiae Hospital, Dublin.

### References

BLACKLEDGE G, LAWTON F, REDMEN C AND KELLY K. (1989). Response of patients in phase II studies of chemotherapy in ovarian cancer: implications for patient treatment and the design of phase II trials. *Br. J. Cancer*, **59**, 650–653.

CANNISTRA SA. (1993). Cancer of the ovary. *N. Engl. J. Med.*, **329**, 1550–1557.

EINZIG AI, WIERNIK PH, SASLOFF J, GARL S, RUNOWICZ C, O'HANLON KA AND GOLDBERG G. (1989). Phase II study of Taxol in patients with advanced ovarian cancer (abstract). *Proc. Am. Soc. Clin. Oncol.*, **8**, 158.

EINZIG AI, WIERNIK PH, SASLOFF J, RUNOWICZ C AND GOLDBERG GL. (1992). Phase II study and long-term follow-up of patients treated with Taxol for advanced ovarian adenocarcinoma. *J. Clin. Oncol.*, **10**, 1748–1753.

GORE ME, FRYATT I, WILTSHAW E AND DAWSON T. (1990). Treatment of relapsed carcinoma of the ovary with cisplatin or carboplatin following initial treatment with these compounds. *Gynecol. Oncol.*, **36**, 707–711.

MARKMAN M, ROTHMAN R, HAKES T, REICHMAN B, HOSKINS W, RUBIN S, JONES W, ALMADRONES L AND LEWIS JL. (1991). Second-line platinum therapy in patients with ovarian cancer previously treated with cisplatin. *J. Clin. Oncol.*, **9**, 389–393.

MCGUIRE WP, ROWINSKY EK, ROSENSHEIN NB, GRUMBINE FC, ETTINGER DS, ARMSTRONG DK AND DONEHOWER RC. (1989). Taxol: A unique anti-neoplastic agent with significant activity in advanced ovarian epithelial neoplasms. *Ann. Intern. Med.*, **3**, 273–279.

MCGUIRE WP, HOSKINS WJ, BRADY MF, KUCERA PR, HOOK KY, PARTRIDGE EE AND DAVIDSON M. (1993). A phase III trial comparing cisplatin/cytoxan (PC) and cisplatin/Taxol (PT) in advanced ovarian cancer (AOC) (abstract). *Proc. Am. Soc. Clin. Oncol.*, **12**, 255.

MCGUIRE WP, HOSKINS WJ, BRADY MF, KUCERA PR, PARTRIDGE EE, HOOK KY AND DAVIDSON M. (1995). Taxol and cisplatin (TP) improves outcome in advanced ovarian cancer (AOC) as compared to cytoxan and cisplatin (CP). (abstract) *Proc. Am. Soc. Clin. Oncol.*, **14**, 771.

SAROSY G, KOHN E, LINK C, ADAM D, DAVIS P, OGNIBENE F, GOLDSPIEL B, CHRISTIAN M AND REED E. (1992). Taxol dose intensification in patients with recurrent ovarian cancer (abstract). *Proc. Am. Soc. Clin. Oncol.*, **11**, 716.

SCHIFF PB, FANT J AND HORWITZ SB. (1979). Promotion of microtubule assembly in vitro by Taxol. *Nature*, **277**, 665–667.

THIGPEN T, BLESSING J, BALL H, HUMMEL S AND BARRETT R. (1990). Phase II trial of taxol as second-line therapy for ovarian carcinoma: A Gynecologic Oncology Group study (abstract). *Proc. Am. Soc. Clin. Oncol.*, **9**, 156.

THIGPEN JT, VANCE RB AND KHANSUR T. (1993). Second-line chemotherapy for recurrent carcinoma of the ovary. *Cancer*, **71**, 1559–1564.

TRIMBLE EL, ADAMS JD, VENA D, HAWKINS MJ, FRIEDMAN MA, FISHERMAN JS, CHRISTIAN MC, CANETTA R, ONETTO N, HAYN R AND ARBUCK SG. (1993). Paclitaxel for platinum-refractory ovarian cancer: results from the first 1000 patients registered to National Cancer Institute treatment referral centre 9103. *J. Clin. Oncol.*, **11**, 2405–2410.